# Tracheoesophageal fistula repair with veno-venous extracorporeal support and Montgomery T-tube: a case report

**DOI:** 10.1051/ject/2025055

**Published:** 2026-03-13

**Authors:** José Luis Che Morales, Gary Kosai Vargas Mendoza

**Affiliations:** 1 Pulmonology and Thoracic Surgery Unit, Medical Center of the Americas Mérida Yucatán México

**Keywords:** Tracheoesophageal fistula, ECMO, Montgomery tube, Airway surgery

## Abstract

Tracheoesophageal fistulas (TEFs) are a major surgical challenge requiring a meticulous approach. This case report describes the successful surgical treatment of a 50 mm TEF extending from the upper third of the trachea to 40 mm above the main carina in a young female patient. The chosen strategy involved an extensive transcervical approach, utilization of Veno-Venous Extracorporeal Membrane Oxygenation (VV-ECMO), and placement of a Montgomery T-tube. The use of ECMO allowed surgical manipulation of mediastinal structures in apnea, ensured adequate gas exchange in the absence of mechanical ventilation, and enhanced comfort during postoperative splinting of the involved structures. The integration of the Montgomery T-tube provided stable airway management postoperatively, particularly advantageous in the context of impaired neurological status precluding early extubation. This combined surgical approach, involving VV-ECMO and Montgomery T-tube, offers a novel, safe, and effective alternative for managing complex TEFs and is recommended for consideration in similar challenging clinical scenarios.

## Overview

ECMO therapy has a wide range of indications, from cardiopulmonary resuscitation to the management of refractory cardiorespiratory failure to its use in elective surgical procedures. One of the earliest published reports on ECMO for airway diseases utilized this technology to treat tracheal stenosis in an infant, subsequently expanding its application to other intrathoracic pathologies [1]. Although ECMO is known to carry a higher risk of bleeding due to the need for anticoagulation [2], this risk can be mitigated by using short-term ECMO with minimal heparinization [3]. In fact, with careful management, we encountered no significant bleeding in this case, highlighting that the benefits of a clear surgical field can be realized without hemorrhagic complications. The surgical team’s improved control over bleeding when using VV-ECMO arises indirectly from several physiological and practical mechanisms rather than from ECMO intrinsically reducing bleeding risks. VV-ECMO indirectly enhances bleeding control during TEF repair by providing a stable, motionless surgical field free from respiratory movements and obstruction by an orotracheal tube. Although ECMO inherently carries a bleeding risk due to anticoagulation, the primary benefit is superior surgical visualization, facilitating precise hemostasis [4].

## Description

**Figure 1:**
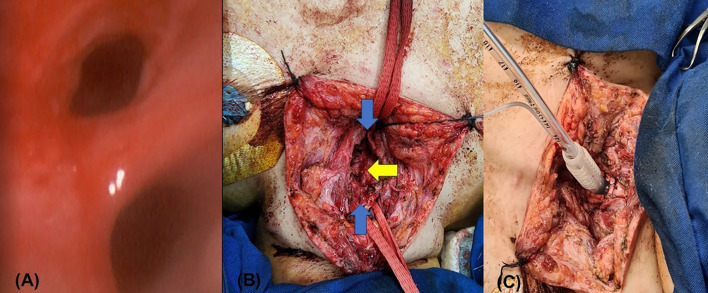
(A): Tracheoesophageal fistula. Esophagus at the bottom. (B): From top to bottom, distal and proximal ends of the trachea (blue arrows). Esophagus with primary closure (yellow arrow). (C): T-Montgomery piece attached to an orotracheal tube for ventilation.

In January 2022, a 25-year-old woman underwent a supratemporal and infratemporal craniectomy for a right petroclival meningioma. Three surgical stages led to a subtotal resection, during which there was significant bleeding. Prolonged mechanical ventilation necessitated a tracheostomy, and a few days later, the cannula yielded food remnants. The bronchoscopy revealed a 10 mm-long tracheoesophageal fistula in the middle third of the trachea, which was conservatively managed with gastrostomy. A week later, she experienced two cardiorespiratory arrests. The first occurred due to a complete obstruction of the cannula, and the second occurred during a new bronchoscopy, which revealed an extension of the fistula up to 50 mm (Figure 1).

The pulmonology-thoracic surgery service at our center thoroughly reviewed the patient’s complex case. After careful consideration of her current condition – including the absence of a diagnosis of brain death and the lack of an ominous prognosis – the team determined that she was a suitable candidate for transfer and surgical intervention at our facility. This decision reflected a multidisciplinary approach focused on optimizing outcomes for patients with challenging airway pathology. The urgency of the surgery stemmed from the need to prevent further aspiration of food material and to ensure the airway’s functionality.

All members of the surgical and critical care team involved in this procedure were certified by the Extracorporeal Life Support Society (ELSO). To provide extracorporeal support during the tracheoesophageal fistula repair, the team implemented veno-venous extracorporeal membrane oxygenation (VV-ECMO) using a femoro-femoral cannulation approach. A multistage left femoral cannula (23 Fr) was utilized for drainage, while a smaller single-lumen cannula (19 Fr) was selected for return. These cannulas were then connected to a Medtronic 560 bioconsole, which managed the ECMO circuit. For gas exchange, an adult Quadrox-i polymethylpentene membrane oxygenator was utilized within the circuit, ensuring efficient oxygenation and carbon dioxide removal throughout the surgical procedure. Cannulation was performed under ultrasound guidance utilizing the Seldinger technique. The drainage cannula was inserted via the right femoral vein, positioning its tip precisely at the junction between the superior vena cava and the right atrium to ensure optimal blood extraction. The return cannula was inserted through the left femoral vein, carefully positioning the distal tip at least 10 cm above the proximal drainage ports, thus optimizing ECMO flow dynamics and minimizing recirculation risk. A single dose of 5000 IU of heparin was administered before surgery and then heparin infusion was initiated at 50 IU/kg/hr, guided by ELSO anticoagulation recommendations, and subsequently titrated according to serial ACT measurements to maintain effective anticoagulation while mitigating bleeding risks [5]. Once adequate oxygenation and acid-base balance were confirmed following the initiation of ECMO, the surgical team proceeded with the removal of the endotracheal tube (ETT). This step allowed the entire procedure to be conducted in a state of apnea, thereby eliminating airway movement and facilitating access to the surgical field. The surgery was performed over a 10-hour period, during which the patient remained apneic, with VV-ECMO providing full respiratory support throughout the operation (Table 1). During the procedure, ECMO flows were carefully titrated to maintain arterial oxygen saturation consistently above 95% and to keep PaCO₂ within normal physiologic limits, thereby ensuring adequate gas exchange and support throughout the period of total apnea. Patient arterial blood gases were obtained at least hourly, with additional samples taken whenever circuit adjustments were made. In addition, intermittent circuit blood gas analyses were performed to verify oxygenator performance and confirm effective extracorporeal gas exchange. Intraoperatively, ECMO management emphasized precise control of oxygenation and ventilation parameters to guarantee cerebral protection in this neurologically compromised patient. This approach differs from bedside ECMO management, which typically prioritizes longer-term hemodynamic stability and anticoagulation balance rather than acute fine-tuning of gas exchange. Circuit parameters were documented every 15 min, with alarm systems in place to detect and immediately signal real-time changes. For safety, cross-matched blood products were prepared and available throughout the operation; in the event of hemorrhagic complications, transfusions would have been delivered via central or peripheral venous access rather than directly into the ECMO circuit. A primed backup ECMO circuit was also maintained on standby to ensure uninterrupted support in the event of emergency circuit failure. The ECMO console was continuously managed by three dedicated team members: an ELSO-certified perfusionist, a cardiothoracic surgeon, and a critical care physician experienced in extracorporeal support.

**Table 1:** Settings, vital signs and blood gases during VV-ECMO run.

	1st hour	5th hour	10th hour
Blood Flow (LPM)	2.6	3.7	3.7
Pump speed (RPM)	1500	2460	2460
Sweep (LPM)	2	1	1
Patient SaO2	100	100	98
Patient PaO2 (mmHg)	311	177	108
Patient PaCO2 (mmHg)	35	35	35
Patient pH	7.46	7.47	7.46
Patient Lactate (mmol/L)	3.1	1.9	1.1
ACT	225	189	186
Heparin infusion (IU/kg/hr)	50	50	50

Abrev. LPM: liters/min; RPM: revolutions/min; FiO2: inspired fraction of oxygen; ACT: activated time of coagulation.

The surgeon carried out a cervicotomy up to the tracheal plane, identifying a 50 mm long TEF and creating a frontal opening through a previous tracheostomy of the 2nd to 5th tracheal rings. The surgeon separated the trachea from the esophagus and performed primary closure in two planes, using a nasogastric tube as a splint. The surgical team interposed bundles of sternocleidomastoid muscle over the esophageal closure and a fibrin sealant. Then, since the back wall of the trachea was absent, a bovine pericardial patch was used to close the defect. A Montgomery tube was then put in as a splint, with one end 7 mm from the vocal cords and the other 15 mm from the main carina. To ensure adequate ventilation, the team cannulated the distal branch of the T tube with a 4.5 Fr tube through the horizontal extremity (Figure 1). Ultimately, the team successfully removed the ECMO device and transferred the patient to the ICU. A chest CT scan performed over one week after the surgery revealed no evidence of TEF. Finally, a month later, the patient returned to her referral hospital, breathing spontaneously through Montgomery’s tube. Six months after the surgery, she was under comprehensive care, experiencing a slow neurological recovery, the family gave consent to communicate the clinical data anonymously for academic purposes.

## Comments

The surgical team’s conduct aligned with current evidence across the three challenges: 1) Access/minimally invasive strategy – an extensive transcervical approach avoided thoracotomy while femoro-femoral VV-ECMO kept the neck free of cannulas, reflecting the principle of selecting the least invasive exposure that still provides secure control of the upper-mid trachea and is described in ECMO-assisted airway cases; 2) Airway manipulation-instituting VV-ECMO and repairing in total apnea eliminated positive-pressure ventilation through the fistula and removed the ETT from the field, matching ELSO-aligned practice and contemporary reviews favoring VV (over VA) for airway surgery; and 3) Postoperative ventilatory support – in the setting of neurological impairment precluding early extubation, use of a Montgomery T-tube provided cuff-less airway splinting, allowed ventilation and secretion management, and protected the repair, consistent with published experience in ventilator-dependent and ECMO-assisted airway scenarios.

In this scenario, the team decided to avoid thoracotomy by making an extensive cervical incision. Given the potential difficulties in managing the distal third of the trachea, the procedure in total apnea with bifemoral VV-ECMO was performed as previously described by other authors [4]. The bifemoral VV-ECMO approach allowed oxygenated blood return similar to the femoro-jugular configuration. This femoro-femoral strategy was specifically chosen for the patient because it kept the neck region completely free of cannulas [6, 7], thereby enabling unobstructed surgical access and optimal manipulation of cervical structures during the procedure, and can provide full pulmonary support without the potential complications of veno-arterial modality [3, 8]. Contemporary guidelines and expert literature recommend VV-ECMO as the preferred approach for airway surgeries, including tracheal and TEF repair procedures, whenever extracorporeal support is indicated [9]. VV-ECMO is favored because it adequately supports gas exchange without the additional risks associated with arterial cannulation, and it is aligned with the principle of using the least invasive ECMO mode required for the patient’s needs [9, 10]. ELSO recommends VV-ECMO for isolated respiratory failure, and recent reviews and case series consistently report routine use of VV-ECMO (often over 80% of cases) in airway operations [9]. VA-ECMO is reserved for the minority of situations where cardiac support or higher perfusion pressures are necessary (for example, coexisting cardiac compromise or certain extensive mediastinal tumors) [10].

As described by Jin et al., significant advantages of Montgomery T-tube usage during ECMO-supported complex airway surgery include stable airway splinting, preservation of airway patency, and facilitation of secretion clearance in patients dependent on prolonged mechanical ventilation [11]. These benefits are supported by previous reports demonstrating effective long-term airway support and secretion drainage capability provided by the Montgomery T-tube in ventilator-dependent and airway stenosis patients [12, 13]. Our clinical experience aligns with these observations, reinforcing the T-tube’s role as an effective airway management tool. The management of an acquired TEF in a patient who cannot be weaned from mechanical ventilation presents a unique challenge. In such cases, standard practice of early extubation or conventional ventilation is often not feasible, as positive-pressure ventilation risks blowing air through the fistula and jeopardizing the surgical repair. This case highlights a novel, multidisciplinary strategy combining veno-venous extracorporeal membrane oxygenation (VV-ECMO) and a Montgomery T-tube to overcome these issues. VV-ECMO can temporarily take over gas exchange, allowing the lungs to be ventilated without positive pressure. This avoids ventilation through the injured airway and protects the fresh TEF repair from barotrauma. In a reported case of a large TEF, mechanical ventilation was deemed technically impossible, and VV-ECMO was used without any need for conventional ventilation [3]. Additionally, by using the T-tube, the team ensured the airway remained secure and the fistula repair was not disrupted by a traditional endotracheal or tracheostomy tube cuff. Notably, Caronia et al. have reported a similar approach where, following endoscopic TEF closure, a T-tube was inserted to protect the suture and maintain the airway patency, resulting in a successfully healed fistula at follow-up [14, 15]. Finally, the use of ECMO did not significantly prolong the surgery; the procedure duration (10 hours) aligns well with previously reported ECMO-supported airway surgeries lasting up to 8 h [3, 16]. Additionally, VV-ECMO allowed oxygenation and ventilation control, ensuring cerebral protection through stable normoxia, normocapnia, and acid-base balance in this neurologically compromised patient as ELSO recommends (Table 1) [17].

To our knowledge, there are no published cases to date that have employed ECMO together with a T-tube for repairing TEF in an adult patient who could not be weaned from the ventilator in the short term due to neurologic sequelae. This outcome suggests that this personalized, multidisciplinary intervention can be replicated to help similar high-risk patients.

## Conclusion

In this 50-mm acquired TEF with neurological impairment, combining femoro-femoral VV-ECMO with a Montgomery T-tube directly addressed the three key challenges – minimizing surgical invasiveness, ensuring safe intraoperative airway control, and securing protected postoperative ventilation. VV-ECMO enabled prolonged apneic surgery with stable gas exchange, eliminating positive-pressure across the fistula and removing the ETT from the field to facilitate an extensive transcervical repair with the neck free of cannulas. The cuff-less Montgomery T-tube then provided a stable airway splint for secretion management and tailored ventilatory support during neurological recovery, avoiding the risks of prolonged intubation and protecting the repair. The uneventful intraoperative course and early radiologic closure support this evidence-aligned, reproducible option for select high-risk adult TEF in experienced centers.

## Data Availability

All pertinent data are included in the manuscript.
